# Perceiving individuality in harpsichord performance

**DOI:** 10.3389/fpsyg.2014.00141

**Published:** 2014-02-24

**Authors:** Réka Koren, Bruno Gingras

**Affiliations:** ^1^Goldsmiths College, University of LondonLondon, UK; ^2^Department of Cognitive Biology, University of ViennaVienna, Austria

**Keywords:** music performance, individuality, harpsichord, categorization, musical expertise

## Abstract

Can listeners recognize the individual characteristics of unfamiliar performers playing two different musical pieces on the harpsichord? Six professional harpsichordists, three prize-winners and three non prize-winners, made two recordings of two pieces from the Baroque period (a variation on a *Partita* by Frescobaldi and a rondo by François Couperin) on an instrument equipped with a MIDI console. Short (8 to 15 s) excerpts from these 24 recordings were subsequently used in a sorting task in which 20 musicians and 20 non-musicians, balanced for gender, listened to these excerpts and grouped together those that they thought had been played by the same performer. Twenty-six participants, including 17 musicians and nine non-musicians, performed significantly better than chance, demonstrating that the excerpts contained sufficient information to enable listeners to recognize the individual characteristics of the performers. The grouping accuracy of musicians was significantly higher than that observed for non-musicians. No significant difference in grouping accuracy was found between prize-winning performers and non-winners or between genders. However, the grouping accuracy was significantly higher for the rondo than for the variation, suggesting that the features of the two pieces differed in a way that affected the listeners’ ability to sort them accurately. Furthermore, only musicians performed above chance level when matching variation excerpts with rondo excerpts, suggesting that accurately assigning recordings of different pieces to their performer may require musical training. Comparisons between the MIDI performance data and the results of the sorting task revealed that tempo and, to a lesser extent, note onset asynchrony were the most important predictors of the perceived distance between performers, and that listeners appeared to rely mostly on a holistic percept of the excerpts rather than on a comparison of note-by-note expressive patterns.

## INTRODUCTION

Identification and categorization are essential features of perception without which it is impossible to properly interpret sensory information (Riesenhuber and Poggio, 2000). Thus, they constitute vital abilities that are crucial for day-to-day survival (Ashby and Maddox, 2005). Generally, the probability that two objects or stimuli will be assigned to the same category or misidentified increases as the similarity between them increases (Ashby and Perrin, 1988). Indeed, there is a tight empirical (Ashby and Lee, 1991) and theoretical (Riesenhuber and Poggio, 2000) link between similarity, categorization, and identification.

The ability to identify individuals is particularly important in species, such as humans, that value kin recognition (Tang-Martinez, 2001) and social interaction (Thompson and Hardee, 2008). The perception of individuality, which is closely related to identification, is in all likelihood also based on a general process of similarity estimation (Ashby and Lee, 1991). Humans are able to identify individuals on the basis of relatively static cues such as facial features (Carey, 1992; Haxby et al., 2000; Andrews and Ewbank, 2004), or by using dynamic displays such as gait and walking (Johansson, 1973; Cutting and Kozlowski, 1977; Loula et al., 2005; Blake and Shiffrar, 2007). This ability to recognize identity cues is not confined to visual perception, but also extends to acoustic cues. Thus, individuals can be recognized and differentiated on the basis of acoustic stimuli such as voices (Belin et al., 2004) – whether those of famous (Van Lancker et al., 1985), familiar (Blatchford and Foulkes, 2006) or unfamiliar people (Sheffert et al., 2002), clapping patterns (Repp, 1987), or even tones which follow similar temporal patterns to clapping (Flach et al., 2004).

Identity cues can also be conveyed efficiently through music performance. Skilled music performance comprises two major components: a technical component and an expressive one (Sloboda, 2000). The former refers mostly to the biomechanical aspects that play a role in producing a fluent performance, while the latter corresponds to intentional variations in performance parameters with the aim of influencing cognitive and esthetic outcomes for the listener. The main performance parameters that can be expressively varied by performers include timbre, pitch, rhythm, tempo, dynamics, and articulation (Sloboda, 2000; Juslin and Sloboda, 2001). Some of these parameters may not be available depending on the musical instrument, and may not be appropriate depending on the musical genre of the piece being performed. Through expressive variations in these parameters, performers not only showcase their musical creativity and personality, but also display their individuality in a manner that may be uniquely identifiable, for instance by playing a well-known repertoire piece in a manner that is recognizably different from typical performances of that piece (Repp, 1992, 1997; Lehmann et al., 2007, p. 85). Hence, famous musicians such as John Coltrane or Sonny Rollins can be recognized after playing only a few notes (Benadon, 2003), and pianists have been shown to be able to recognize their own performances from modified recordings in which only temporal information was available (Repp and Knoblich, 2004; Repp and Keller, 2010).

However, few studies have investigated whether listeners could accurately distinguish between unfamiliar performers playing different interpretations of the same piece on the same instrument. We have recently shown that both non-musicians and musicians perform significantly above chance in such a task, even in the absence of timbral or dynamic differentiation, supporting previous findings which suggested that timing cues can be sufficient to enable performer recognition (Gingras et al., 2011). Our results also showed that expressive interpretations were sorted more accurately than inexpressive ones. Although these findings indicate that listeners can distinguish between unfamiliar performers playing two different interpretations of the same piece, it remained to be seen whether similar results would be observed in a study using performances from two different pieces. Using a machine-learning approach, Stamatatos and Widmer (2005) had previously shown that a set of classifiers trained on a database of piano performances of 22 pianists playing one piece by Fryderyk Chopin could reliably recognize these same pianists performing a different piece by the same composer. Indeed, the authors noted that the 70% recognition rate achieved by their learning ensemble represented “a level of accuracy unlikely to be matched by human listeners” (Stamatatos and Widmer, 2005, p. 54), a claim that has not been empirically verified so far to our knowledge and that stands in contrast to earlier observations suggesting that humans are generally more accurate than machines in such categorization tasks (Ashby and Maddox, 1992).

The main objective of the present study was to test whether human listeners are indeed able to recognize unfamiliar performers playing two different pieces, by asking listeners to group together excerpts from two different harpsichord pieces which they think have been played by the same performer. Although our study does not use the same stimuli and design as Stamatatos and Widmer (2005), it can be considered a general test of their prediction that human listeners cannot match the accuracy of a learning ensemble in such a task. Thus, the current study differs from our earlier study (Gingras et al., 2011) in its use of two different pieces instead of one piece recorded with two different interpretations, in addition to its focus on harpsichord instead of organ music. As indicated by previous research (Repp and Knoblich, 2004; Gingras et al., 2011), excerpts from the same piece can be matched to the same performer by relying mostly on expressive timing and articulation patterns. However, successfully matching excerpts from two different pieces would presumably require the listener to detect identity cues of a more general nature that transcend specific pieces, such as performer-specific timing or articulation patterns that can be found across different pieces (see Gingras et al., 2013). We therefore hypothesized that matching excerpts from the same piece should be perceptually easier than matching excerpts from different pieces, and that this would be reflected in a higher level of accuracy. We also considered the possibility that some pieces would be easier to sort accurately than others, either because they afford performers more possibilities for conveying their artistic individuality, or because they can be processed more easily by listeners.

Our rationale for using harpsichord performances in this study was to extend performance research on other keyboard instruments besides the piano, as there is a large body of research on piano performance (see Gabrielsson, 2003 for a review), but few published empirical studies on harpsichord performance. Moreover, the harpsichord is not widely known in the general public, and thus represents an ideal medium for a study on the recognition of unfamiliar performers. Finally, its mechanism affords no or very little timbre differentiation (excluding registration changes), and only limited dynamic variation (Penttinen, 2006). Consequently, as with organ performance (Gingras, 2008; Gingras et al., 2010), expressivity in harpsichord performance is confined mostly to timing and articulation. Because all recordings were realized on the same instrument and with the same registration (configuration of stops controlling the timbre), listeners had to rely almost exclusively on temporal parameters to discriminate between performers, as in our earlier study (Gingras et al., 2011).

Another aim of the study was to investigate the effects of the performer’s level of expertise and the effects of musical training on categorization accuracy. Repp (1997) showed that recordings from world-famous pianists tend to be perceived by listeners as exhibiting more individuality than those of graduate students in piano performance. This link between the performers’ level of expertise and their perceived individuality was also observed in the study by Gingras et al. (2011), which showed that listeners sorted performances by prize-winning performers more accurately than those by non-prize-winners.

Several studies have demonstrated a timbre- and pitch-processing advantage for musicians versus non-musicians (for a review, see Chartrand et al., 2008). However, the effect of musical expertise appears to be task-dependent, and a number of responses to musical stimuli are largely unaffected by musical training (Bigand and Poulin-Charronnat, 2006). Both musicians and non-musicians can reliably distinguish among different levels of expressiveness in performances of the same piece (Kendall and Carterette, 1990), and discriminate between familiar and novel performances of the same piece (Palmer et al., 2001). Although musicians discriminated between performers more accurately than non-musicians in the sorting task described in Gingras et al. (2011), the difference was not statistically significant. Here, we also compared the sorting accuracy of musicians and non-musicians by using an experimental design similar to our earlier study. Additionally, we also controlled for possible gender effects by balancing the number of male and female participants for both musicians and non-musicians.

As in Gingras et al. (2011), we used a constrained sorting task in which participants are given information about the underlying category structure (in this case, the number of performers and the number of pieces) prior to the experiment. Whereas unconstrained sorting tasks tend to focus on the processes leading to category construction, constrained tasks focus on the types of category structures that can be learned by participants in the absence of trial-by-trial feedback (Ell and Ashby, 2012) and are thus appropriate for investigating listeners’ ability to discriminate between individual performers.

## METHODS

### RECORDING HARPSICHORD PERFORMANCES

#### Participants

Twelve professional harpsichordists, five female and seven male, from the Montreal (Canada) area recorded the pieces that were used as stimuli in the listening experiment. Their average age was 39 years (range: 21–61 years). They had played the harpsichord for a mean duration of 22 years (range: 6–40). Seven of them had previously won prizes in regional, national, or international harpsichord competitions. Ten reported being right-handed, one left-handed, and one ambidextrous. All harpsichordists signed a consent form and received financial compensation for their participation in the study, which was approved and reviewed by the Research Ethics Board of McGill University (Montreal, QC, Canada).

#### Materials

Two pieces were selected for this study: the third variation from the *Partita No. 12 sopra l’aria di Ruggiero* by Girolamo Frescobaldi (1583–1643), and *Les Bergeries*, a rondo by François Couperin (1668–1733). Both pieces are representative of the Baroque harpsichord repertoire.

#### Procedure

For the *Bergeries*, performers received no instructions besides playing the pieces as if in a “recital setting.” Each piece was recorded twice. In the case of the *Partita*, performers were instructed to play three versions, each emphasizing a different voice (respectively, the soprano, alto, and tenor parts). Each of the three versions was recorded twice, for a total of six recordings per performer. The order of the instructions for the *Partita *was randomized according to a Latin square design. The entire recording session lasted approximately one hour. Only the recordings emphasizing the highest voice were used here.

Performances took place in an acoustically treated studio, on an Italian-style Bigaud harpsichord (Heugel, Paris, France) with two 8-foot stops. Only the back stop was used for the experiment. This harpsichord was equipped with a MIDI console, allowing precise measurement of performance parameters. MIDI velocities were estimated by a mechanical double contact located underneath the keys and from which the travel time of the keys was measured, with a high velocity corresponding to a shorter travel time (faster attack). MIDI velocity values for each note event were coded in a range between 16 (slowest) and 100 (fastest). The measured velocities were calibrated separately for each key prior to the recording sessions.

The audio signal was recorded through two omnidirectional microphones MKH 8020 (Sennheiser GmbH, Wedemark Wennebostel, Germany). The microphones were located 1 m above the resonance board and were placed 25 cm apart. The audio and MIDI signals were sent to a PC computer through an RME Fireface audio interface (Audio AG, Haimhausen, Germany). Audio and MIDI data were then recorded using Cakewalk’s SONAR software (Cakewalk, Inc., Boston, MA, USA) and stored on a hard disk.

### SORTING TASK

#### Participants

Twenty participants with two or fewer years of musical training (mean age = 27.4 years, SD = 7.2 years), henceforth referred to as non-musicians, and 20 participants having completed at least 1 year in an undergraduate university in music performance or musicology (mean age = 30.2 years, SD = 9.6 years), henceforth referred to as musicians, participated in the experiment. Both groups of participants were balanced for gender. All participants signed a consent form and received a small gift for their participation in the study and the chance to win one out of four prizes worth 20 pounds each. The study was approved by the Research Ethics Board of Goldsmiths College, University of London (London, UK).

#### Materials

**FIGURE 1:**
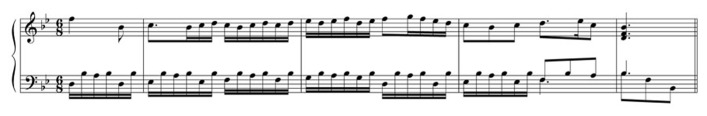
**Excerpt from Couperin’s *Bergeries *used in the sorting task**.

**FIGURE 2:**
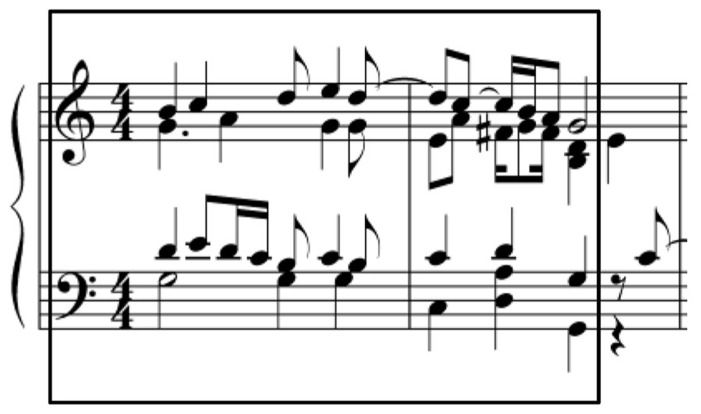
**Excerpt from Frescobaldi’s *Partita *used in the sorting task.** The boxed section corresponds to the fragment heard by participants.

In order to reduce the number of excerpts to a manageable number, four performances (two for each piece) from three prize-winners and three non-prize-winners were used for the sorting task, for a total of 24 excerpts. The six performers were selected according to the following criteria: small error rate (few wrong or missing notes), small tempo differences between both performances of the same piece, and overall quality of the recordings. From the *Bergeries*, an excerpt corresponding to the end of the rondo section was chosen (**Figure 1**), whereas the beginning of the *Partita *was retained (**Figure 2**). Both excerpts were chosen to be syntactically coherent musical units with a clear harmonic closure (perfect authentic cadence) at the end. The duration of the excerpts was 11– 15 s for the *Bergeries *excerpts, and 8–11 s for the *Partita *excerpts. Audacity was used for cutting out and editing (fading in and fading out) the excerpts. The task was practiced with a reduced training stimulus set using four excerpts (two for each piece) from two different performers whose recordings were not included in the main experiment, for a total of eight excerpts.

#### Procedure

The experimental interface, programmed in MATLAB (Gingras et al., 2011), consisted of a computer monitor on which the musical excerpts were represented by 24 randomly numbered icons which when clicked on played the corresponding audio recordings. Participants could listen to the excerpts in any order and as many times they wished. They were asked to group together the excerpts that they believed to have been played by the same performer by moving them into one of six boxes that represented the six performers. The icons corresponding to each of the two pieces had different colors and participants were told that each performer had played each piece twice. Participants had to listen to an excerpt at least once before being able to drag its corresponding icon into a box representing a performer. At the end of the experiment participants were required to listen to the content of each box before finishing the experiment, to ensure that they had listened to each excerpt at least twice. For the experiment to be completed, each box had to contain exactly two icons corresponding to each piece. There was no time limit for the categorization but the time taken to arrange the selections was recorded. Prior to the sorting task, participants practiced the task in a familiarization phase using eight alternate excerpts played by two performers whose recordings were not included in the main task. No feedback was provided on the participants’ performance in the familiarization phase. The experiment took place in a sound-attenuated booth. Participants wore Sennheiser HD202 headphones and were screened for peripheral hearing problems by a standard audiometric procedure, using an Amplivox 2160 pure-tone diagnostic audiometer prior to testing. After finishing the computer-based sorting task, participants were asked to complete a questionnaire about their musical background and the strategies they used to complete the sorting task.

### PERFORMANCE DATA ANALYSIS

Performances were matched to the scores of the pieces using an algorithm developed by Gingras and McAdams (2011). Four expressive parameters were analyzed for each excerpt: articulation, note onset asynchrony, timing, and velocity. Articulation refers to the amount of overlap between two consecutive note events *n*_i_ and *n*_j_ belonging to the same melodic line or voice. A legato articulation corresponds to a positive overlap (when the offset of note *n*_i_ occurs after the onset of note *n*_j_), whereas a detached or staccato articulation corresponds to a negative overlap. Here, the onset of a note is defined as the time at which the corresponding key is pressed (as measured by the MIDI system) and its offset corresponds to the time at which the key is released. Note onset asynchrony is defined as the difference in onset time between note onsets that are notated in the musical score as synchronous (Palmer, 1989). To analyze expressive timing, tempo values were computed from the inter-onset interval (IOI) between consecutive note onset events. In the case of velocity, the raw MIDI velocity values associated with the key press corresponding to each note onset were used for the analysis. In addition, performance errors (wrong notes and missing notes) were also identified using the score-performance matcher described in Gingras and McAdams (2011).

## RESULTS

### ANALYSIS OF THE EXPRESSIVE PARAMETERS OF THE PERFORMANCES

In order to compare the excerpts on the basis of their expressive parameters, an analysis was conducted on the following parameters: mean tempo (expressed as mean dotted quarter-note duration in the case of the *Bergeries *and mean quarter-note duration in the case of the *Partita*), tempo variability [expressed as the coefficient of variation of the tempo, which corresponds to the standard deviation of the (dotted) quarter-note duration normalized by the mean duration], articulation (expressed as the degree of overlap between successive notes), and onset asynchrony (referring to the difference in onset times between notes that are attacked simultaneously in the score, such as notes belonging to the same chord). These parameters essentially comprise the range of expressive factors that are controlled by the performer in harpsichord music (excluding registration effects, which were controlled for in this experiment). **Table 1** lists the mean values for these parameters averaged over both recordings of each piece for each performer (identified by the letters A to F). Because performance errors could also potentially contribute to identifying individual performers, **Table 1** also includes the total number of performance errors for each recording.

**Table 1:** Mean values for the expressive parameters and total performance errors for each performer.

Performer	A*	B	C	D*	E	F*
**Bergeries**
Mean dotted quarter-note duration (ms)	1361 (3)	1701 (40)	1293 (39)	1513 (16)	1572 (28)	1630 (9)
Mean coefficient of variation of dotted quarter-note duration (%)	6.7 (2.8)	14.8 (0.1)	11.1 (1.3)	20.9 (0.3)	10.0 (1.5)	12.1 (0.1)
Mean overlap (% of note duration)	29.3 (3.0)	23.3 (1.5)	31.4 (6.8)	4.0 (0.7)	8.1 (1.0)	10.0 (1.3)
Mean root-mean-square asynchrony (ms)	15 (4)	36 (1)	31 (6)	23 (1)	6 (3)	15 (1)
Mean velocity (MIDI units)	59 (0)	51 (0)	58 (0)	58 (1)	63 (0)	60 (1)
Total performance errors (for each recording)	0; 0	0; 1	4; 2	0; 1	0; 0	0; 0
***Partita***
Mean quarter-note duration (ms)	1272 (19)	1287 (22)	1236 (50)	1474 (13)	1144 (28)	1218 (0)
Mean coefficient of variation of quarter-note duration (%)	6.1 (1.6)	11.5 (1.1)	7.0 (0.1)	12.0 (0.1)	10.4 (3.9)	7.0 (0.3)
Mean overlap (% of note duration)	-2.0 (2.6)	2.3 (1.7)	-12.1 (3.2)	-14.4 (1.7)	-14.6 (1.6)	-7.8 (1.2)
Mean root-mean-square asynchrony (ms)	29 (1)	55 (5)	23 (3)	66 (8)	40 (13)	33 (1)
Mean velocity (MIDI units)	48 (0)	60 (2)	55 (1)	57 (0)	63 (4)	54 (2)
Total performance errors (for each recording)	1; 2	0; 0	1; 1	0; 0	1; 0	0; 0

*Prize-winners are indicated with an asterisk. Standard deviations of the mean values obtained for each of the two recordings per performer are given in parentheses.*

Because the purpose of this analysis was to investigate whether there were significant differences between performers, mixed-model analyses of variance were conducted for each of the five aforementioned expressive parameters, with performer as a random factor and order of recording as a fixed factor, on the 12 excerpts from each piece that were used in the sorting task (**Table 2**). No statistical analyses were conducted on the performance errors, given that the majority of recordings did not contain a single error. The order of recording was not a significant factor for any of the expressive parameters (all *p*-values > 0.2). The significance of the random effects associated with each performer was assessed by comparing a model that incorporated only the fixed effect of the order of the recording to a model that also included a random intercept associated with each performer.

**Table 2:** Mixed-models analyses of variance on the expressive parameters.

Piece	***Bergeries***	***Partita***
Mean tempo	*χ*^2^(1) = 14.10, *p* < 0.001 B*, C*	*χ*^2^(1) = 10.31, *p* = 0.001 D**
Coefficient of variation of the tempo (%)	*χ*^2^(1) = 8.93, *p* = 0.003 A*, D*	*χ*^2^(1) = 2.46, *p* = 0.117 n.s.
Mean overlap (% of note duration)	*χ*^2^(1) = 10.55, *p* = 0.001 C*, D*	*χ*^2^(1) = 8.40, *p* = 0.004 B*
Mean root-mean-square asynchrony (ms)	*χ*^2^(1) = 9.00, *p* = 0.003 B*, E*	*χ*^2^(1) = 5.80, *p* = 0.016 D*
Mean velocity (MIDI units)	*χ*^2^(1) = 17.55, *p* < 0.001 B**, E*	*χ*^2^(1) = 7.16, *p* = 0.008 A*, E*

Individual performers are identified by codes A to F. The significance of the random intercept effects predicted for each individual performer was assessed using two-tailed *t*-tests. *p < 0.05; **p < 0.01; n.s.: no significant effect.

The IOI was computed for each dotted quarter note for all *Bergeries *excerpts, and for each quarter note for all *Partita *excerpts. The mean value obtained for each excerpt was used as a measure of tempo. Significant differences in mean quarter-note duration were observed between performers, with performers B being significantly slower and C significantly faster for the *Bergeries*, and D being significantly slower for the *Partita*.

The coefficient of variation of the tempo, obtained by dividing the standard deviation of the tempo by the mean tempo and expressing the result as a percentage of the mean (dotted) quarter-note duration, was used as a measure of the degree of tempo variability. Whereas significant differences between performers were observed for the *Bergeries*, with performer A displaying a smaller amount of variation and performer D a larger one, no significant differences were found for the *Partita*.

Articulation refers to the amount of overlap between two consecutive note events *n*_i_ and *n*_j_ belonging to the same melodic line or voice. A positive overlap indicates a *legato* articulation, while a negative value represents a detached or *staccato* articulation. Because the amount of overlap varies with tempo (Repp, 1995), we chose to use the overlap ratio, defined as the ratio of the overlap between two consecutive note events and the IOI between these notes, as a measure of articulation (Bresin and Battel, 2000). Significant differences in the amount of overlap were found between performers for the *Bergeries*, with performer C playing more *legato* and D playing more detached, and for the *Partita*, with performer B playing more *legato*.

Note onset asynchrony is defined as the difference in onset time between note onsets that are notated in the musical score as synchronous (Palmer, 1989). Several measures of onset asynchrony have been constructed. Rasch (1979) proposed to use the root mean square, or standard deviation of the onset times of nominally simultaneous notes. We chose to use this measure here. Significant differences were observed between performers for the *Bergeries*, with performer B using larger asynchronies and performer E using smaller ones, and for the *Partita*, with performer D using larger asynchronies. Asynchronies were generally larger than those observed in organ performance (Gingras et al., 2011), and comparable to or even larger than the asynchronies of 15–20 ms which are typically observed in piano performance (Palmer, 1989). Given that the reported threshold for detecting onset asynchronies is around 20 ms (Hirsh, 1959), listeners could conceivably differentiate between performers on the basis of the amount of onset asynchrony.

Finally, the mean MIDI velocity associated with the keypress corresponding to each note onset was computed for both excerpts. Significant differences were observed between performers for the *Bergeries*, with performer B using lower velocities and performer E using higher ones, and for the *Partita*, with performer A using lower velocities and performer E again using higher ones.

From these analyses, we may conclude that performers could be statistically differentiated on the basis of mean tempo, mean overlap, amount of onset asynchrony, and velocity for both pieces, and additionally on the basis of the amount of variation of the tempo in the case of the *Bergeries*.

### GENERAL ASSESSMENT OF THE CATEGORIZATION ACCURACY

To assess the categorization accuracy for each participant, we compared their partitioning of the excerpts with the correct categorization solution, which corresponds to a grouping of the 24 excerpts in which all excerpts played by the same performer are grouped together and no excerpts played by different performers are grouped together. Categorization accuracy was evaluated using the adjusted Rand index (Hubert and Arabie, 1985), a chance-corrected measure of the agreement between the correct categorization solution and the grouping proposed by the participant. A positive adjusted Rand index indicates that a greater number of excerpts were grouped correctly than would be expected by chance (“chance” corresponding here to a randomly generated partition of the excerpts), whereas a negative adjusted Rand index indicates that fewer excerpts were grouped correctly than would be expected by chance, and a value of zero corresponds to chance performance. 39 participants (out of 40) performed better than chance (corresponding to a positive adjusted Rand index), with only one non-musician performing worse than chance (corresponding to a negative adjusted Rand index). Furthermore, for 17 musicians (85%) and nine non-musicians (45%), the adjusted Rand index was significantly above zero (indicating a performance significantly better than chance), one-tailed *p *< 0.05 estimated using a bootstrapped (Efron and Tibshirani, 1993) null distribution of 1,000,000 permutations with replacement with a mean adjusted Rand index of -0.0001, 95% CI [-0.101, 0.133]. The difference between the proportion of musicians and non-musicians who performed significantly better than chance was significant, as determined by a chi-square test, *χ*^2^(1) = 7.03, *p* = 0.008. The same proportion of male and female participants, 65% (corresponding to 13 participants for each gender) performed significantly better than chance.

To evaluate the effect of musical training and gender on sorting accuracy, we conducted an analysis of variance with musical training and gender as between-subject factors. Variances did not deviate significantly from homogeneity, as indicated by a Levene test, and the distribution of the adjusted Rand indices did not deviate from normality across factorial combinations. Musicians performed significantly better than non-musicians, *F*(1,36) = 15.527, *p* < 0.001, *η*^2^ = 0.301. The effect of gender was not significant, *F*(1,36) = 0.110, *p* = 0.742, *η*^2^ = 0.002, and no significant interaction was found between musical training and gender, *F*(1,36) = 0.002; *p* = 0.962, *η*^2^ = 0.000.

Listening activity, defined by the total number of times a participant listened to the excerpts, was found to be significantly correlated with categorization accuracy, *r*(38) = 0.480, *p* = 0.002. The correlation was stronger for musicians, *r*(18) = 0.458, *p* = 0.043, than for non-musicians, *r*(18) = 0.332, *p* = 0.152, but this difference was not significant as determined by a *Z*-test on the Fisher-transformed correlation coefficients (*z* = 0.43, *p* = 0.664). Very similar results were obtained when correlating the total amount of time spent on the sorting task with the categorization accuracy (the total amount of time spent on the task was highly correlated with listening activity, *r*(38) = 0.988, *p* < 0.001). Although musicians listened to more excerpts than non-musicians on average, the difference was not statistically significant, as shown by a two-tailed Mann-Whitney test (the data was not normally distributed), *U* = 168.0, *p* = 0.394.

### EFFECT OF PIECE AND PERFORMER EXPERTISE ON CATEGORIZATION ACCURACY

**FIGURE 3:**
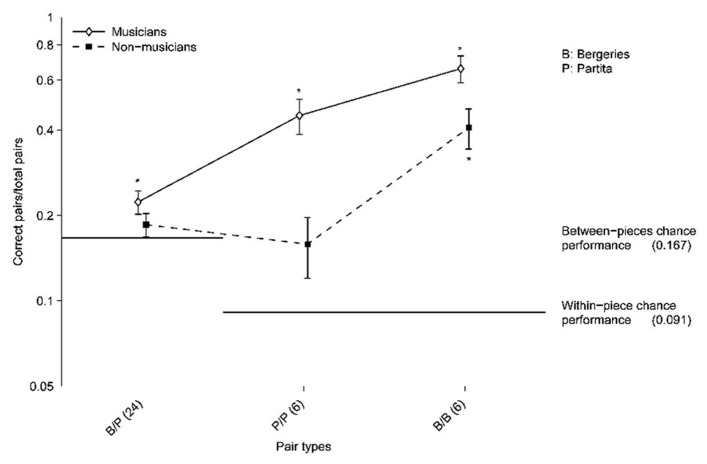
**Proportion of correct pairs compared to the total number of pairs for all pair types.** The total number of pairs is indicated in parentheses for each pair type. B/P: pairs corresponding to one *Bergeries* and one *Partita* excerpt from the same performer. P/P: pairs corresponding to two *Partita* excerpts from the same performer. B/B: pairs corresponding to two *Bergeries* excerpts from the same performer (B/B). Error bars indicate standard errors of the mean. Asterisks indicate values that are significantly different from chance performance (for both musicians and non-musicians) as determined by one-tailed *t*-tests with Bonferroni correction. Points are connected to help distinguish the two participants group visually.

To assess whether the ability of participants to correctly sort excerpts varied according to the piece, and to compare the participants’ ability to correctly group together excerpts from the same performer and the same piece versus same performer/different pieces, the participants’ partitions were decomposed by taking into account the performers and the pieces corresponding to the excerpts that were grouped together. Such analyses involve comparisons of pairs of excerpts (Miller, 1969; Daws, 1996). The proportion of pairs of excerpts correctly grouped together (pairs played by the same performer and identified as such) out of the total number of pairs was then computed for the following types of pairs (**Figure 3**):

(a) One *Bergeries* and one *Partita* excerpt from the same performer (B/P).
(b) Two *Partita* excerpts from the same performer (P/P).
(c) Two *Bergeries* excerpts from the same performer (B/B).

Combinatorial probabilities are used to determine the chance performance level. In the present case, a partition of 24 excerpts into six groups of four excerpts yields 36 pairs of excerpts, given by 6 × [4!/(2! × 2!)]. The total number of possible pairs is given by 24!/(22! × 2!), yielding 276 pairs. These 276 pairs can be further decomposed in 66 possible pairs comprising two *Bergeries *excerpts, given by 12!/(10! × 2!), 66 possible pairs comprising two *Partita *excerpts, and the remaining 144 pairs which contain one excerpt from each piece.

To compute the chance performance level, we need to estimate the probability of randomly assigning a pair to a given partition. Here, the chance performance level, which corresponds to the probability of randomly assigning a pair to a given partition, differs between pairs containing one excerpt from each piece and pairs comprising two excerpts from the same piece. Because participants were constrained to assign exactly two *Bergeries *excerpts and two *Partita *excerpts to each performer, each partition always included exactly six pairs comprising two excerpts from the *Bergeries*, six pairs comprising two excerpts from the *Partita*, and 24 pairs comprising one excerpt from each piece (note that four such combinations are formed when assigning two *Bergeries *excerpts and two *Partita *excerpts to a performer, hence the 24 pairs obtained for the six performers). Therefore, the chance performance level for same-piece pairs is equivalent to 6/66, or *p = *0.091, whereas the chance performance level for *Bergeries–Partita* pairs is equivalent to 24/144, or *p* = 0.167.

The proportion of correct pairs was significantly above chance for *Bergeries–Bergeries *pairs, for both musicians and non-musicians, as determined by one-tailed *t*-tests with Bonferroni correction (the distributions did not deviate significantly from normality). However, only musicians performed significantly above chance in the case of the *Partita–Partita *pairs and in the case of the *Bergeries–Partita *pairs. Note that the *t*-test for the *Partita–Partita *pairs for non-musicians barely reached significance (*p* = 0.043) prior to the Bonferroni correction.

Because chance performance levels depend on the pair composition, further analyses comparing across pair types were conducted on the chance-corrected proportions of correct pairs. Additionally, in order to estimate the effect of the performers’ expertise (prize-winners versus non-prize-winners) on the participants’ performance in the sorting task, the categorization accuracy for the prize-winners was compared with that observed for the non-prize-winners. Furthermore, because listening activity was significantly correlated with categorization accuracy, it was included as a covariate in subsequent analyses. Gender was excluded because previous analyses had indicated that it was not a significant factor. Thus, a repeated-measures logistic regression analysis on the chance-corrected proportion of correct pairs was conducted, with participants’ musical training as a between-subjects factor, performer expertise and pair composition (*Bergeries–Bergeries*, *Partita–Partita*, and *Bergeries–Partita*) as within-subject factors, and listening activity as a continuous covariate associated with each participant. Chance performance for each pair type was added to the model as an offset, following the procedure described in Lipsitz et al. (2003). We verified that the only parameter estimate that was affected by adding this offset to the logistic regression model was the main effect of pair composition. In line with prior analyses, significant effects were observed for musical training, *χ*^2^(1) = 5.72, *p* = 0.017, pair composition, *χ*^2^(2) = 483.02, *p* < 0.001, and listening activity, *χ*^2^(1) = 7.45, *p* = 0.006. In addition, a significant interaction between musical training and pair composition was found, *χ*^2^(2) = 16.28, *p* < 0.001, which corresponds to the larger effect of musical training observed on the *Partita–Partita* pairs compared to the *Bergeries–Bergeries* pairs (**Figure 3**). However, performer expertise was not a significant predictor of the chance-corrected proportion of correct pairs, *χ*^2^(1) = 0.09, *p* = 0.763. No other interactions reached significance (all *p*-values > 0.25).

*Post hoc* tests using the Bonferroni correction procedure were used to compare the chance-corrected proportion of correct pairs for the three different types of pairs, collapsing over levels of musical training. All pairwise comparisons were significant, indicating that the chance-corrected accuracy for *Bergeries–Bergeries *pairs was significantly higher than for the other two pair types, and that the accuracy for *Partita–Partita* pairs was significantly higher than for *Bergeries–Partita *pairs, all Bonferroni-corrected *p*-values < 0.001.

### PERCEPTUAL DISTANCE BETWEEN THE EXCERPTS AND PERFORMANCE PARAMETERS

In order to assess whether performance parameters such as articulation, asynchrony, tempo, or velocity could explain the perceived distance between excerpts, with perceptually distant excerpts corresponding to excerpts rarely or never grouped together and “close” excerpts (excerpts thought to have been played by the same performer) representing excerpts always grouped together, we conducted a distance-based multivariate regression using forward selection (McArdle and Anderson, 2001). This analysis seeks to model the proportion of variance in the perceptual distance between excerpts (obtained from the co-occurrence matrix) that is explained by the mean values of the performance parameters obtained for each excerpt (see **Table 1**). Because significance testing for distance-based multivariate regression is done through a permutation procedure, degrees of freedom are not reported. Furthermore, the *F*-ratios yielded by this procedure do not exactly correspond to the *F*-ratios obtained in a traditional analysis of variance and are thus labeled “pseudo-*F* ratios.” We used the DISTLM-forward program (Anderson, 2003) to conduct these analyses. The forward selection procedure used the proportion of the variance explained by each expressive parameter as criterion for selection. For all analyses, 99,999 permutations were conducted to test for statistical significance. **Table 3** reports the results of the distance-based multivariate regression analyses conducted separately for each piece and between both pieces.

**Table 3:** Distance-based multivariate regression.

	Marginal tests			Sequential tests		
	Pseudo-*F*	Variance	*p*	Pseudo-*F*	Variance	*p*
***Bergeries***
Mean tempo	2.53	0.202	<0.001	2.53	0.202 (1)	<0.001
Coefficient of variation of the tempo (%)	2.01	0.167	0.007	2.12	0.152 (2)	0.023
Mean overlap (% of note duration)	2.04	0.170	0.007	0.78	0.051 (4)	0.586
Mean root-mean-square asynchrony (ms)	1.66	0.142	0.061	2.23	0.141 (3)	0.036
Mean velocity (MIDI units)*	1.74	0.149	0.052	N/A	N/A	N/A
**Total variance explained by significant predictors in the forward-stepwise model: 0.495**
***Partita***
Mean tempo	2.25	0.184	<0.001	2.14	0.140 (3)	0.004
Coefficient of variation of the tempo (%)*	1.95	0.163	0.006	N/A	N/A	N/A
Mean overlap (% of note duration)	1.67	0.143	0.024	1.95	0.144 (2)	0.003
Mean root-mean-square asynchrony (ms)	2.36	0.191	<0.001	2.36	0.191 (1)	<0.001
Mean velocity (MIDI units)	1.88	0.158	0.004	1.41	0.088 (4)	0.150
**Total variance explained by significant predictors in the forward-stepwise model: 0.476**
**Between pieces**
Mean tempo	2.12	0.088	<0.001	2.12	0.088 (1)	<0.001
Coefficient of variation of the tempo (%)	1.22	0.052	0.158	1.02	0.040 (4)	0.494
Mean overlap (% of note duration)	1.09	0.047	0.341	1.33	0.052 (3)	0.074
Mean root-mean-square asynchrony (ms)	2.03	0.085	<0.001	2.06	0.082 (2)	<0.001
Mean velocity (MIDI units)	0.31	0.014	0.998	0.30	0.012 (5)	0.992
**Total variance explained by significant predictors in the forward-stepwise model: 0.170**

*Marginal tests correspond to tests conducted separately on each expressive parameter taken in isolation. Sequential tests represent the relative contribution of each parameter after taking into account other parameters already included in the model, based on a forward selection procedure using the proportion of variance explained by each parameter as the criterion for selection (the order of entry is given in parentheses). Parameters marked with an asterisk were not included in the sequential tests due to multicollinearity.*

Although the influence of multicollinearity on the model results cannot be evaluated directly when using the DISTLM procedure (Link et al., 2013), highly correlated independent variables can lead to spurious results about their relationships with the dependent variable (Zar, 1999). Thus, performance parameters that were highly correlated (∣ *r*∣ > 0.7) were not included in the forward-selection model (Zar, 1999), although they were included in the marginal tests because we cannot exclude the possibility that these parameters play a role in the listeners’ evaluation of perceptual distance simply due to the presence of multicollinearity (see **Table 4** for the correlation matrices). For each pair of highly correlated variables, the variable with the highest correlations with the remaining independent variables was excluded from the forward-selection procedure. In the case of the *Bergeries*, velocity was strongly inversely correlated with asynchrony, *r*(10) = -0.86, *p *< 0.001, and was excluded. For the *Partita*, the coefficient of variation of the tempo was highly correlated with both asynchrony, *r*(10) = 0.67, *p *= 0.017, and velocity, *r*(10) = 0.77, *p *= 0.003, and was excluded. No other performance parameters were highly correlated.

**Table 4:** Correlation matrices on the mean values for the expressive parameters.

	Mean tempo	Coefficient of variation of the tempo (%)	Mean overlap (% of note duration)	Mean root-mean-square asynchrony (ms)
**Bergeries**
Mean tempo	1			
Coefficient of variation of the tempo (%)	0.37	1		
Mean overlap (% of note duration)	-0.53	-0.54	1	
Mean root-mean-square asynchrony (ms)	0.00	0.41	0.48	1
Mean velocity (MIDI units)	-0.29	-0.34	-0.38	-0.86
**Partita**
Mean tempo	1			
Coefficient of variation of the tempo (%)	0.41	1		
Mean overlap (% of note duration)	-0.02	-0.17	1	
Mean root-mean-square asynchrony (ms)	0.64	0.67	0.02	1
Mean velocity (MIDI units)	-0.15	0.77	-0.34	0.39

*Correlations computed on the mean values for each expressive parameter for each excerpt.*

In the case of the *Bergeries*, tempo, tempo variation, and overlap were significant predictors of the perceptual distance between excerpts according to the marginal tests. Tempo, tempo variation, and asynchrony were significant predictors in the forward selection model (sequential tests). Separate analyses for musicians and non-musicians were also conducted to examine potential differences between groups (not shown in **Table 3**). The results for musicians were similar to the results on the entire group of participants, whereas asynchrony was also significant in the marginal tests for non-musicians, and the forward selection model included only tempo and asynchrony as significant predictors.

In the case of the *Partita*, all parameters were significant predictors of the perceptual distance between excerpts according to the marginal tests. Asynchrony, overlap, and tempo were significant predictors in the forward selection model. The results for musicians on the marginal tests were similar to the results on the entire group of participants, but the forward selection model included overlap, asynchrony, and velocity as significant predictors. All parameters except overlap were significant in the marginal tests for non-musicians, whereas tempo, asynchrony, and overlap were significant in the forward selection model.

To evaluate the relationship between the perceptual distance between both pieces and the performance parameters, standardized values (*z*-scores) were used for the parameters. Moreover, the distances for all within-piece comparisons were set to chance performance, thus leaving the sum of the distance matrix elements unchanged but confining the variance to the between-pieces quadrants (note that all distance-based regression models are based on square, symmetrical distance matrices and thus require some type of algebraic manipulation in order to enable the type of between-pieces comparison conducted here; similar problems arise with related methods such as redundancy analysis or non-metric multidimensional scaling). Tempo and asynchrony were significantly correlated with perceived distance according to marginal tests, and both parameters were significant predictors in the forward selection model. Similar results were obtained for musicians and non-musicians. The proportion of variance explained by the forward-stepwise model on all participants was considerably smaller (0.170) than that explained by the models considering only one piece (respectively 0.495 for the *Bergeries *and 0.476 for the *Partita*), in line with the observation that participants performed on average barely above chance in this situation (especially in the case of non-musicians).

The analyses conducted in this section have, until now, focused solely on the mean values for the performance parameters, computed over an entire excerpt. However, it is also plausible that listeners would pay attention to note-by-note (or event-by-event) expressive profiles, and that two excerpts with similar profiles would be judged as more likely to have been played by the same performer. The magnitude of the correlations between the expressive profiles corresponding to different performers can be used to evaluate the degree of similarity between these profiles. Hence, following the method outlined in Gingras et al. (2013), we computed Kendall’s tau correlations between all pairs of performers for each piece and for each performance parameter (to avoid pseudoreplication, the values for the two recordings associated with each piece were averaged before computing the correlations). Four performance parameters were considered: tempo, overlap, asynchrony, and velocity. The correlation matrices thus obtained for each parameter were used as similarity matrices. Mantel tests were conducted to evaluate the degree of similarity between the similarity matrices corresponding to the expressive profiles associated with each performance parameter on the one hand, and the co-occurrence matrix corresponding to the perceptual distance between excerpts as judged by listeners on the other hand. The statistical significance of the Mantel tests was assessed using the Bonferroni correction procedure.

In the case of the *Bergeries*, the similarity matrices corresponding to the expressive profiles did not correlate significantly with the perceptual distance between excerpts, with the exception of asynchrony. However, although the uncorrected *p-*value for the asynchrony matrix was significant (*p *= 0.014 before the Bonferroni adjustment), the correlation was negative (Mantel *r* = -0.52), which means that this association was probably an artifact of another relationship as it is unlikely that listeners would group together excerpts whose asynchrony profiles differed markedly. Similar results were obtained for both musicians and non-musicians.

In the case of the *Partita*, no correlation between the similarity matrices corresponding to the expressive profiles and the co-occurrence matrix reached significance (all uncorrected *p*-values > 0.2). Similar results were observed for both musicians and non-musicians. These results suggest that, for either the *Bergeries* or the *Partita*, listeners did not rely on the degree of similarity between note-by-note expressive profiles when grouping excerpts together.

## DISCUSSION

This study examined whether listeners are able to accurately group together short excerpts from two different harpsichord pieces (Couperin’s *Bergeries *and Frescobaldi’s *Partita*) played by the same performer, while taking into consideration both performer and listener expertise. Although most participants reported that they experienced the sorting task as being very difficult, an analysis of the categorization accuracy of individual participants revealed that 39 of 40 participants performed above chance, with 26 participants at a level significantly better than chance. As in earlier work by Gingras et al. (2011), musicians performed better than non-musicians on the sorting task, but in this case the difference between the two groups was significant, whereas it did not reach significance in the previous study. This result overlaps with Davidson’s (1993) findings that music students performed significantly better than non-musicians when asked to distinguish between the performance manners of different pianists and violinists, suggesting that musical training has an important role in recognizing personal characteristics in a short musical excerpt. More generally, our findings are in line with Ashby and Maddox’s (1992) observations that novices are generally less accurate than experienced categorizers.

The influence of musical training may have been stronger here than in Gingras et al. (2011) due to the fact that the participants had to compare two different pieces here, instead of two different interpretations of the same piece as in the earlier study. Indeed, whereas both musicians and non-musicians performed significantly better than chance on the *Bergeries–Bergeries *pairings, only musicians performed significantly better than chance when considering the *Partita–Partita *or *Bergeries–Partita *pairings. These findings suggest that the effect of musical training may be stronger with some pieces or musical styles, and that successfully matching excerpts from two different pieces to the same performer may require extensive musical training. The musicians’ familiarity with specific musical cues may have had a positive impact on their performance on the task. Moreover, musicians may also have a better ability to retain the characteristics of the excerpts in memory, although this remains to be evaluated. Commitment to the task (as shown by the increased amount of time spent listening to the excerpts) was also shown to be a good predictor of the participants’ performance, replicating the results reported in Gingras et al. (2011). However, gender was not related to the ability to perceive artistic individuality. Indeed, male and female participants performed very similarly on average.

We hypothesized that there would be an effect of performers’ level of musical expertise on the listeners’ grouping accuracy, that is, excerpts played by prize-winning performers would be easier to group accurately than excerpts played by non-prize-winners. This assumption was based on earlier results in a similar task (Gingras et al., 2011). However, our results did not show any significant effect of performers’ expertise. It is possible that the consistency and distinctiveness of the performers, which were associated with the level of expertise in Gingras et al. (2011), were not as relevant here, especially since two different pieces were compared.

Because the two pieces selected for this experiment differed in tempo, melody, texture, duration, and meter, we considered the possibility that excerpts from one piece might be easier or more difficult to sort accurately than excerpts from the other piece. Indeed, the results suggested that there was a significant difference between the two pieces in terms of grouping accuracy, the *Bergeries* excerpts proving to be sorted more accurately. The question of interest here is explaining what made one piece more easily recognizable than the other. Although the results presented here do not provide a direct answer to that question, they do provide some plausible interpretations. As shown in **Table 2**, significant differences between performers could be found for all five expressive parameters analyzed here in the case of the *Bergeries*, whereas only four parameters yielded significant differences between performers for the *Partita*. Moreover, at least two performers were significantly different from the mean for each parameter in the case of the *Bergeries*, suggesting the possibility to differentiate perceptually between these performers based on the parameter in question, in contrast to the *Partita *where in most cases only one performer was significantly different from the mean. Another explanation for the better grouping accuracy observed for the *Bergeries* may be simply that the *Bergeries *excerpts contained more notes (75 versus 37 for the *Partita*) and were a few seconds longer on average than the *Partita *excerpts (11–15 s for the *Bergeries *versus 8–11 s for the *Partita*), thus giving participants more time to recognize the distinctive features of an excerpt. Although the fact that the excerpts did not have exactly the same length for both pieces may be considered a potential impediment when comparing the performance on the two pieces, we deemed it important to select stimuli that represented complete musical units with a sense of closure, and the excerpts chosen likely constituted the most appropriate selection in that regard. A further explanation for the difference in sorting accuracy between both pieces could be the greater distinctiveness of the *Bergeries *fragment selected for the experiment, with its lighter texture, regular rhythm, and clear melody, making it *a priori* easier to process than the corresponding *Partita *fragment, with its more complex polyphonic texture. In that regard, it is noteworthy that the difference in sorting accuracy observed between musicians and non-musicians was much more manifest in the case of the *Partita *than for the *Bergeries*, suggesting that non-musicians were especially affected by the difference in texture between the two pieces. However, it should be noted that both non-musicians and musicians performed above chance in a comparable task using an excerpt of organ music whose length, number of notes, style, complexity, and polyphonic texture were very similar to that of the *Partita* (compare **Figure 2** with **Figure 1** in Gingras et al., 2011).

We were also interested in examining whether listeners fared better in matching the excerpts from the same pieces (either the *Bergeries* or the *Partita*) or the excerpts from different pieces but played by the same performer. A significant difference in the sorting accuracy was found between grouping the excerpts from the same piece and grouping excerpts from different pieces (in addition to the difference observed between the *Bergeries *and the *Partita *described above) after correcting for chance performance, as indicated by *post hoc* tests. Moreover, only musicians performed significantly above chance when considering only pairings of excerpts from different pieces. These results indicate that participants were not very successful in matching excerpts from different pieces, especially in the case of non-musicians. Nevertheless, the fact that musicians could perform above chance in this situation suggests that some distinctive features associated with a performer’s specific playing style can be recognized across different pieces, even in the case of unfamiliar performers (thus extending the work of Benadon, 2003 on famous performers) and on an instrument limiting the use of expressive strategies associated with timbre and dynamics. These results are in line with the findings reported in Gingras et al. (2013), and with the earlier work by Stamatatos and Widmer (2005) using an artificial intelligence approach. Although a direct comparison with the results reported by Stamatatos and Widmer is not possible due to the different experimental design, our results suggest that these authors were apparently correct to note that human listeners were not likely to match the accuracy of a learning ensemble when attempting to sort performances of two different pieces by their performer.

Additionally, we investigated the relationship between performance parameters and the perceptual distance between performers as established from the results of the sorting task using distance-based multivariate regression analysis. Although the resulting regression models differed between pieces, as well as between musicians and non-musicians in some cases, some overarching conclusions could nevertheless be gleaned from these analyses. First, we note that marginal tests for tempo were significant in all analyses, and that tempo entered practically all forward-stepwise models (the only exception being the *Partita *in the case of musicians). The fact that tempo was used prominently by listeners in such a task is in line with earlier results (Repp and Knoblich, 2004; Gingras et al., 2011). Second, non-musicians appeared to rely on note onset asynchrony to a greater extent than musicians: asynchrony entered all models for non-musicians, but was only a significant predictor in the case of the *Partita *and the between-pieces comparisons for musicians. Third, tempo and asynchrony were the only significant predictors of perceptual distance when comparing between pieces, for both musicians and non-musicians. This suggests that listeners found it difficult to rely on other expressive features such as overlap, velocity, or tempo variation when comparing across different pieces. In the case of tempo variation, the fact that the two pieces were written in a different meter, apart from the considerable textural differences, may explain why listeners did not rely on this parameter. On the other hand, in the case of velocity, some performers were consistent across both pieces (performer E, for instance, used significantly higher velocities than other performers in both pieces). However, it is possible that these differences in velocity, which lead to contrasts in sound intensity amounting to a few dB at most on the harpsichord (Penttinen, 2006), could not be easily perceived by listeners. Although overlap entered a few regression models, it appeared to be a generally secondary parameter, especially in comparison to organ performance where overlap was, along with tempo, a major predictor of perceptual distance between performers (Gingras et al., 2011). This may be partially explained by the fact that the harpsichord sound decays relatively quickly, at least in comparison to other keyboard instruments such as the piano or organ, thus making the contrast between *legato *and *staccato *articulation less striking on this instrument. Finally, correlational analyses between note-by-note expressive patterns and perceptual distances (as obtained from the sorting task) suggested that listeners did not appear to rely on note-by-note patterns in the sorting task. This leads us to surmise that they relied mostly on a holistic impression of the excerpts, which is captured in a rough manner by the distance-based multivariate regression models based on the mean values computed for each of the expressive parameters analyzed here.

Because performance errors could also conceivably contribute to the identification of individual performers, we listed the total number of performance errors for each recording in **Table 1**. As can be seen, the error totals were very low. Most of these errors (11 of 14) consisted in omissions, meaning that a note present in the score was not played. Such “silent” errors are likely to be inconspicuous and, as shown by Repp (1996), most performance errors are typically difficult to detect, even for trained musicians. Additionally, none of these errors occurred in the highest voice (or part), causing them to be less noticeable (Palmer and Holleran, 1994). Indeed, author Bruno Gingras, a trained musicologist, could not detect most of these performance errors even when listening to the recordings while following with the score (note that participants in the sorting task did not have access to the score of the pieces). For these reasons, it is very unlikely that performance errors could have been used reliably by listeners to discriminate between performers. One performer (harpsichordist C) committed a somewhat larger number of errors in the *Bergeries *excerpts, but all of these errors consisted in omissions in the left-hand part (lower voice) and were thus not conspicuous.

In conclusion, very few studies have so far investigated the ability of humans to process identity cues in music performance, especially with unfamiliar performers. To our knowledge, the present study is the first empirical study that investigated the participants’ ability to accurately discriminate between unfamiliar performers playing excerpts from two different pieces. The study by Gingras et al. (2011), on which the current work was modeled, served as a good benchmark for comparison. Both studies showed that most participants, both musicians and non-musicians, are able to recognize and process identity cues in short musical excerpts (of approximately 10–15 s in both studies) and to correctly group excerpts that are played by the same performer at a level better than chance. Both studies also showed that sorting accuracy was significantly correlated with the time spent doing the sorting task. Although musicians performed better than non-musicians in both studies, the effect only reached significance in the present case. However, whereas Gingras et al. (2011) reported an effect of performer expertise, no such effect was observed here. Moreover, the present study underscored that the choice of musical excerpts may exert an important influence on the sorting accuracy. Nevertheless, the fact that these studies yielded generally similar results, even though the experiments were conducted using a different instrument and stylistic repertoire, in addition to being carried out in different countries, suggest that the findings are indeed valid and reliable. Overall, our results indicate that the performers’ expertise may not be as essential in predicting individual recognition as is the musical background of the participants and the characteristics of the excerpts when more than one piece is involved (however, the influence of the performers’ expertise should ideally be evaluated using a larger group of performers, as well as other measures of expertise besides performance prizes). Moreover, our findings suggest that specific features associated with a piece may play a crucial role in enabling listeners to pick up on its characteristics and recognize the identity of the performer, and that extensive musical training may be a prerequisite for perceiving identity cues across different pieces, at least in the case of short excerpts played by unfamiliar performers.

## Conflict of Interest Statement

The authors declare that the research was conducted in the absence of any commercial or financial relationships that could be construed as a potential conflict of interest.
